# METACASPASE9 modulates autophagy to confine cell death to the target cells during *Arabidopsis* vascular xylem differentiation

**DOI:** 10.1242/bio.015529

**Published:** 2016-01-06

**Authors:** Sacha Escamez, Domenique André, Bo Zhang, Benjamin Bollhöner, Edouard Pesquet, Hannele Tuominen

**Affiliations:** Umeå Plant Science Centre, Department of Plant Physiology, Umeå University, Umeå 90187, Sweden

**Keywords:** *Arabidopsis thaliana*, Autophagy, Intercellular signalling, Metacaspase, Programmed cell death, Tracheary element

## Abstract

We uncovered that the level of autophagy in plant cells undergoing programmed cell death determines the fate of the surrounding cells. Our approach consisted of using *Arabidopsis thaliana* cell cultures capable of differentiating into two different cell types: vascular tracheary elements (TEs) that undergo programmed cell death (PCD) and protoplast autolysis, and parenchymatic non-TEs that remain alive. The TE cell type displayed higher levels of autophagy when expression of the TE-specific *METACASPASE9* (*MC9*) was reduced using RNAi (*MC9*-RNAi). Misregulation of autophagy in the *MC9*-RNAi TEs coincided with ectopic death of the non-TEs, implying the existence of an autophagy-dependent intercellular signalling from within the TEs towards the non-TEs. Viability of the non-TEs was restored when *AUTOPHAGY2* (*ATG2*) was downregulated specifically in *MC9*-RNAi TEs, demonstrating the importance of autophagy in the spatial confinement of cell death. Our results suggest that other eukaryotic cells undergoing PCD might also need to tightly regulate their level of autophagy to avoid detrimental consequences for the surrounding cells.

## Abstract (precis)

**Summary:** In cell cultures that simulate *Arabidopsis* xylem differentiation, METACASPASE9 modulates the level of autophagy during programmed cell death to prevent ectopic death of the surrounding cells.

## INTRODUCTION

The development of multicellular organisms involves programmed cell death (PCD). In land plants, PCD – followed by complete cellular autolysis – likely represented the first evolutionary step in the acquisition of specialized water-conducting structures that were necessary for survival in the dry atmosphere (Escamez and Tuominen, 2014; Friedman and Cook, 2000). Nowadays higher plants possess an efficient vascular tissue called xylem (Brodribb, 2009; Kenrick and Crane, 1997), in which the water-conducting tracheary element (TE) cells deposit cellulosic patterned secondary cell walls (SCW) before cell death (Oda and Fukuda, 2012; Oda et al., 2010; Pesquet et al., 2010; Torrey et al., 1971). TE cell death is brought about by rupture of the central vacuole (Burgess and Linstead, 1984; Groover and Jones, 1999; Kuriyama, 1999), which is believed to allow TE autolysis to occur by releasing certain hydrolytic enzymes and by activating others when the acidic contents of the vacuole leak into the cytoplasm (Bollhöner et al., 2013; Groover and Jones, 1999; Wodzicki and Brown, 1973). No molecular effectors of TE cell death have been identified, but TE *post-mortem* autolysis has been shown to involve specific hydrolases (Avci et al., 2008; Ito and Fukuda, 2002) including *Arabidopsis thaliana* METACASPASE9 (MC9) (Bollhöner et al., 2013).

Metacaspases (MCs) are cysteine proteases that are structurally related to metazoan caspases (Uren et al., 2000). Plant metacaspases are divided into two classes; type I, which contains enzymes with a prodomain consisting of both a proline-rich domain and a zinc finger, and type II, which contains MC family members without any prodomain. Besides the type II Arabidopsis MC9 which fosters TE *post-mortem* autolysis, several metacaspases have been shown to play a role in different plant cell types undergoing PCD (Bozhkov et al., 2005; Coll et al., 2010; He et al., 2008; Minina et al., 2013; Watanabe and Lam, 2011). In particular, the cells undergoing PCD in spruce somatic embryos express a type II MC which functions upstream of autophagy (Minina et al., 2013). Autophagy is a trafficking route commonly used by cells for various purposes such as recycling of the cellular contents during starvation (Mizushima et al., 2004; Mortimore and Poso, 1987; Thompson et al., 2005) and cellular differentiation (Alvarez et al., 2008; Kwon et al., 2010; Mizushima and Levine, 2010). However its role in the regulation of cell death has been debated (Lv et al., 2014). For example, the normal progression of PCD in spruce embryos requires metacaspase controlled autophagy, although the cell death program itself is not executed by autophagy (Minina et al., 2013). Minina et al. (2013) also proposed that other plant cell types undergoing PCD could utilize a similar process of metacaspase-regulated autophagy.

Autophagy has been claimed to play a crucial role in the progression of TE PCD (Kwon et al., 2010). However, no published study has been able to determine whether TEs require autophagy to execute PCD or whether autophagy is merely required to promote TE differentiation. Furthermore, numerous studies on autophagy rely on mutants with increased or suppressed autophagy in all cell types, which does not allow identification of specific regulators and functions of autophagy in a particular cell type. In the case of TEs, the function of autophagy remains poorly understood and a potential relation between autophagy and MCs has not been investigated. We therefore hypothesized the existence of a link between MC9 and autophagy during TE differentiation. To test this hypothesis, we utilized an *Arabidopsis thaliana in vitro* TE cell culture, which allows detailed and specific characterization of TE differentiation without interference from the other tissue types. In these cell cultures, hormonal stimulus is used to induce part of the cells to differentiate into TEs, while the other cells – hereafter called non-TEs – stay alive (Pesquet et al., 2010). With the help of this system we could observe that correct regulation of autophagy by MC9 in TEs is required for spatial confinement of cell death.

Abbreviations:

ATG2: AUTOPHAGY2
cLSM: confocal laser scanning microscopy
DIC: differential interference contrast
EXO70: exocyst subunit 70
FDA: fluorescein diacetate
GFP: green fluorescent protein
GRI: GRIM REAPER
GUS: β-glucuronidase
IRX1: IRREGULAR XYLEM1
MC: metacaspase
MC9: METACASPASE9
MS: Murashige and Skoog medium
PCD: programmed cell death
PCR: polymerase chain reaction
PI: propidium iodide
qPCR: real-time quantitative PCR
SCW: secondary cell wall
s.d.: standard deviation
TE: tracheary element

## RESULTS

### MC9 is involved in TE differentiation in cell cultures

**Fig. 1.**
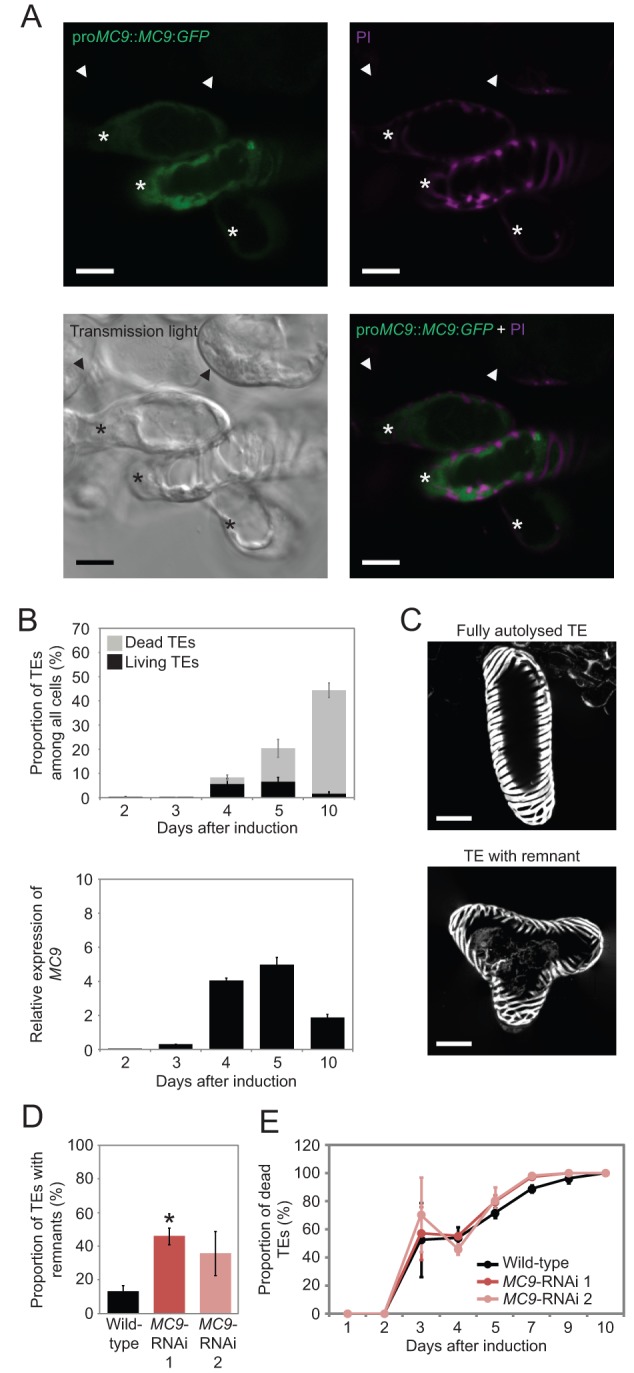
**MC9 is involved in TE differentiation in cell suspensions.** (A) cLSM micrographs of pro*MC9*::*MC9*:GFP cell cultures stained with propidium iodide (PI, cell wall stain, magenta) five days after induction. Asterisks indicate TEs and arrowheads indicate non-TEs. Scale bars=10 µm. (B) Proportions of living and dead TEs (top) and *MC9* transcript abundance measured by qPCR (bottom) during TE differentiation (*n*=3). (C) 3D projection of cLSM micrographs showing the middle part of PI-stained wild-type TEs to exemplify autolysed (top) and remnant-containing (bottom) TEs ten days after induction of TE differentiation. Note that in addition to staining cell walls, PI also stains protoplast remnants after cell death. Scale bars=20 µm. (D) Proportion of TEs (among all TEs) containing a remnant in wild-type and two *MC9*-RNAi lines ten days after induction of TE differentiation (*n*=3). (E) Proportion of dead TEs (among all TEs) during TE differentiation in wild-type and two *MC9*-RNAi lines (*n*=3). Error bars show mean±s.d.; *P<0.05.

We first investigated whether MC9 is expressed in differentiating TEs *in vitro* as it is *in planta* (Bollhöner et al., 2013). Thus, we expressed a MC9:GFP fusion protein under the transcriptional control of *MC9* promoter (pro*MC9*::*MC9*:*GFP*) in differentiating TE cell cultures. Consistent with *in planta* data (Bollhöner et al., 2013), microscopy analysis of three transgenic lines revealed that MC9:GFP was specifically expressed in TEs, recognizable by their patterned SCWs (Fig. 1A). Furthermore, *MC9* transcript levels corresponded to the proportion of living TEs in differentiating cell cultures (Fig. 1B).

Next, we created cell lines downregulated for *MC9* using a constitutive 35S promoter driven RNAi construct (hereafter *MC9*-RNAi) in order to functionally characterize the role of MC9 during *in vitro* TE differentiation. At the fifth day of TE differentiation, we measured *MC9* transcript levels in order to select two independent *MC9*-RNAi lines (*MC9*-RNAi 1 and 2) with reduced *MC9* expression (Fig. S1A,B). At the end of the differentiation, the TEs of *MC9*-RNAi lines retained remnants of undegraded protoplast more often than wild-type TEs (Fig. 1C,D). However, the timing and extent of TE cell death remained unaffected in the *MC9*-RNAi TE cell cultures (Fig. 1E). Therefore, MC9 promotes *post-mortem* autolysis during *in vitro* TE differentiation, as it does in whole plants (Bollhöner et al., 2013).

### The TE-specific MC9 prevents ectopic death of the surrounding cells by influencing intercellular signalling

**Fig. 2.**
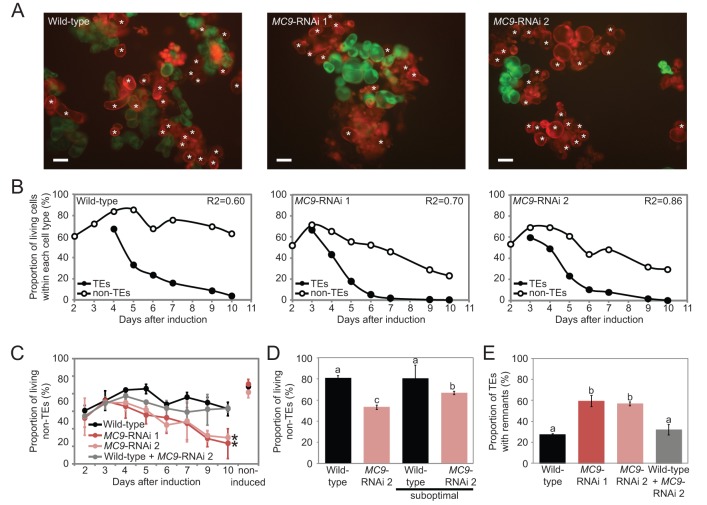
**Downregulation of *MC9* influences intercellular signalling during TE differentiation.** (A) Fluorescence micrographs of wild-type (left) and two *MC9*-RNAi cell lines (middle and right) stained with FDA (viability, green) and PI (cell walls, red) 10 days after induction. Asterisks indicate TEs. Scale bars=50 µm. (B) Proportions of living TEs (among all TEs) and non-TEs (among all non-TEs) during TE differentiation in wild-type and two *MC9*-RNAi lines. R2 indicates the square Pearson's correlation coefficient between the both aforementioned variables (*n*=3). (C) Proportion of living non-TEs (among all non-TEs) during TE differentiation in wild-type and two *MC9*-RNAi lines and in a 1:1 mix of wild-type and *MC9*-RNAi 2 cells. Cell viability in the non-induced conditions after ten days demonstrates that ectopic cell death occurs in the *MC9*-RNAi cells only upon induction of TE differentiation (*n*=3). (D) Proportions of living non-TEs (among all non-TEs) in wild-type and *MC9*-RNAi 2 lines ten days after being induced to differentiate with normal or suboptimal hormone levels (resulting in ∼50% reduction of TE differentiation) (*n*=3). (E) Proportion of TEs (among all TEs) containing a remnant in wild-type, two *MC9*-RNAi lines and in a 1:1 mix of wild-type and *MC9*-RNAi 2 cells ten days after induction of TE differentiation (*n*=3). Error bars show mean±s.d.; *P<0.05 in C; means that do not share any letter are significantly different (*P*<0.05) in D,E.

When staining the differentiating cell suspensions with the viability dye fluorescein diacetate (FDA), we observed that the *MC9*-RNAi cell cultures displayed many dead non-TE cells, whereas the wild-type non-TEs remained viable (Fig. 2A-D). This ectopic death of the non-TEs was unexpected because *MC9* is not expressed in this cell type (Fig. 1A) (Bollhöner et al., 2013), which implies the existence of intercellular signalling between TEs and non-TEs and that MC9 influences this process.

The ectopic non-TE death correlated temporally with TE PCD in *MC9*-RNAi lines (Fig. 2B), suggesting that the dying TEs had become detrimental for the survival of the non-TEs. This hypothesis was further supported by restoration of the non-TE viability in *MC9*-RNAi cell cultures when the proportion of TEs was purposely reduced by half (Fig. 2D) or when mixing equal proportions of wild-type and *MC9*-RNAi cells (Fig. 2C). In the latter experiments, also TE autolysis was partially restored (Fig. 2E). Therefore, MC9 seems to be involved in processes that influence all or part of the signalling between TEs and non-TEs, which normally fosters TE autolysis and prevents ectopic death of the non-TEs.

### MC9 modulates autophagy in TEs

Restriction of cell death to the target cell type during plant PCD has been hypothesized to rely on autophagy (Bozhkov and Jansson, 2007). Interestingly, autophagy was previously shown to be regulated by a metacaspase in spruce embryo suspensor cells undergoing PCD (Minina et al., 2013). We therefore examined whether MC9 could restrict cell death to TEs by modulating autophagy. Increased autophagy in the differentiating *MC9*-RNAi cells was suggested by the lower abundance of non-degraded autophagy cargo NBR1 compared with the wild-type cells (Fig. S2). However, the cell type in which autophagy is altered in the *MC9*-RNAi cell cultures cannot be determined from the above observations because they rely on protein extracts from cell populations containing both TEs and non-TEs.

**Fig. 3.**
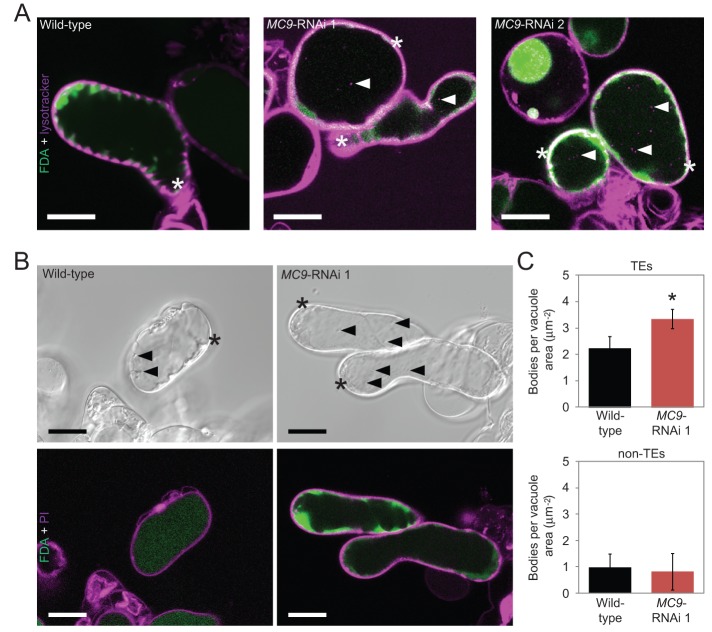
**MC9 regulates autophagy specifically in TEs.** (A) cLSM micrographs of differentiating cells stained with FDA (viability, green) for viability and with lysotracker (magenta) for acidic bodies in the vacuole. Asterisks indicate living TEs and arrowheads indicate examples of lysotracker-stained vacuolar bodies. Scale bars=20 µm. (B) DIC micrographs (top) and cLSM micrographs (bottom) of wild-type and *MC9*-RNAi 1 cells treated with Concanamycin A and stained with FDA (viability, green) and PI (cell walls, magenta) five days after induction. Asterisks indicate living TEs and arrowheads indicate example of vacuolar bodies in the displayed focal plane. Scale bars=20 µm. (C) Abundance of vacuolar bodies in TEs (top) and non-TEs (bottom) of Concanamycin A-treated wild-type and *MC9*-RNAi 1 lines, five days after induction of TE differentiation (*n*≥3). Error bars show mean±s.d.; *P<0.05.

In order to measure autophagy in a cell-specific manner, we undertook two different microscopy approaches to analyse accumulation of autophagic bodies in TEs as well as in the non-TEs. First, differentiating cell cultures were stained with the acidotropic dye Lysotracker Red DN99 (hereafter lysotracker), which stains vacuolar autophagic bodies. Analyses by confocal laser scanning microscopy (cLSM) suggested higher abundance of lysotracker-stained bodies in the vacuoles of *MC9*-RNAi TEs than in the wild-type TEs (Fig. 3A). However, lysotracker did not allow precise quantification of the vacuolar bodies due to unspecific staining of the cell walls. Quantification of the vacuolar bodies was achieved with our second approach which consisted of differential interference contrast (DIC) microscopy after treatment of the cells with Concanamycin A, which inhibits degradation of the vacuolar contents and hence leads to accumulation of the vacuolar bodies (Klionsky et al., 2012; Merkulova et al., 2014; Yoshimoto et al., 2004). Consistent with the decreased NBR1 abundance (Fig. S2) and with the increased abundance of lysotracker-stained bodies in *MC9*-RNAi TEs (Fig. 3A), we detected more bodies by DIC microscopy in the vacuoles of the *MC9*-RNAi TEs than in the wild-type TEs (Fig. 3B,C). These vacuolar bodies in TEs likely represented autophagic bodies because simultaneous treatment with the autophagy inhibitor wortmannin (Blommaart et al., 1997) reduced their number (Fig. 5A). The number of vacuolar bodies did not differ between non-TEs of the different lines (Fig. 3C), supporting the fact that MC9 modulates autophagy specifically in TEs.

**Fig. 4.**
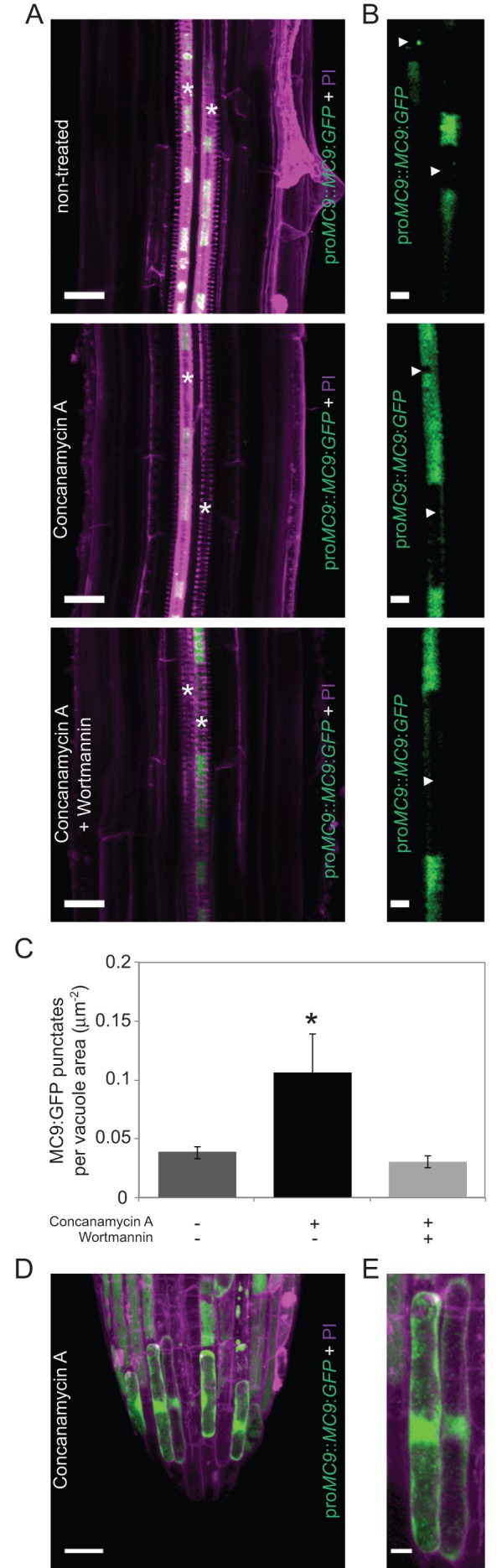
**MC9:GFP is present in the vacuole of cells undergoing PCD.** (A) 3D projections of cLSM micrographs of a PI-stained (cell walls, magenta) pro*MC9*::*MC9*:*GFP* (green) seedling's root treated or not with Concanamycin A and without or with wortmannin. Asterisks indicate TEs. Scale bars=20 µm. (B) Blow up of the GFP signal in single focal planes from (A) used for vacuolar GFP-punctate quantification (C). Vacuoles are indicated by white arrowheads. Scale bars=5 µm. (C) Quantification of vacuolar MC9:GFP punctates in TEs of pro*MC9*::*MC9*:*GFP* seedling's root treated or not with Concanamycin A and without or with wortmannin (*n*=3). Error bars show mean±s.d.; *P<0.05. (D) 3D projection of cLSM micrographs of the lateral root cap of a PI-stained pro*MC9*::*MC9*:*GFP* seedling treated with Concanamycin A. Scale bar=20 µm. (E) Blow up of two cells from (D). Scale bar=5 µm.

Modulation of TE autophagy by MC9 implies that MC9 functions prior to cell death, which is contradictory to the current view that MC9 is solely cytoplasmic and can only be activated at the time of cell death when the cytoplasmic pH drops (Bollhöner et al., 2013). To determine whether MC9 could also localize to the vacuole of differentiating TEs, and thus act *pre-mortem*, we analysed pro*MC9*::*MC9*:GFP seedlings treated with Concanamycin A which prevents, in addition to vacuolar degradation, quenching of GFP in the vacuole. cLSM analyses revealed the presence of MC9:GFP in TEs and lateral root cap cells (Fig. 4A-E), two cell types undergoing PCD in roots (Bollhöner et al., 2013; Fendrych et al., 2014). MC9:GFP was mainly localized in the cytoplasm but a clear punctate localization was also observed in the vacuoles (Fig. 4B-E). Interestingly, the MC9:GFP-positive vacuolar punctates were reminiscent of autophagic bodies and their number was significantly reduced when inhibiting autophagy by wortmannin treatment (Fig. 4A-C). In conclusion, even though a majority of MC9 is localized in the cytoplasm, MC9 is also present in the vacuole of differentiating TEs, possibly associated with autophagic bodies.

### Spatial confinement of vascular cell death is dependent on the level of autophagy in TEs

**Fig. 5.**
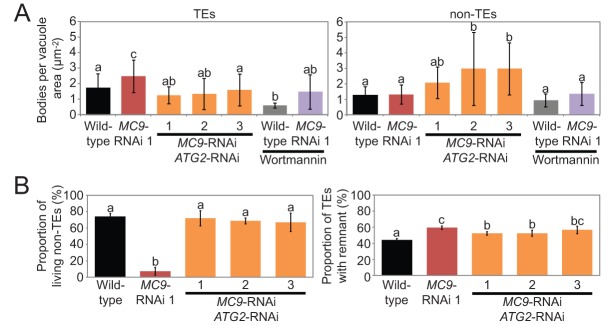
**Autophagy in TEs controls ectopic cell death and TE autolysis downstream of MC9.** (A) Abundance of vacuolar bodies in TEs (left) and non-TEs (right) of Concanamycin A-treated cell cultures five days after induction of TE differentiation in wild-type (*n*=11 for TEs and *n*=13 for non-TEs), *MC9*-RNAi 1 (*n*=15 for TEs and *n*=11 for non-TEs) and *MC9*-RNAi pro*IRX1*::*ATG2*-RNAi line 1 (*n*=12 for TEs and *n*=13 for non-TEs), 2 (*n*=7 for TEs and *n*=8 for non-TEs) and 3 (*n*=9 for TEs and *n*=11 for non-TEs), as well as in wild-type (*n*=6 for TEs and *n*=6 for non-TEs) and *MC9*-RNAi 1 (*n*=8 for TEs and *n*=7 for non-TEs) simultaneously treated with the autophagy inhibitor wortmannin. (B) Proportions of living non-TEs (among all non-TEs; left) and of TEs with remnants (among all TEs; right) in wild-type, *MC9*-RNAi 1 and three *MC9*-RNAi pro*IRX1*::*ATG2*-RNAi lines ten days after induction of TE differentiation (*n*=3). Error bars show mean±s.d. Means that do not share any letter are significantly different (*P*<0.05).

To test whether increased autophagy could explain the ectopic cell death and the impaired TE autolysis of the *MC9*-RNAi lines, autophagy was specifically suppressed in TEs of the *MC9*-RNAi line 1. We generated an RNAi construct against the autophagy gene *ATG2* which is known to be a relevant target to efficiently modify autophagy in plants (Hackenberg et al., 2013; Inoue et al., 2006). The *ATG2*-RNAi construct was placed under the control of the TE-specific promoter of *IRREGULAR XYLEM1*/*CELLULOSE SYNTHASE8* (*IRX1*/*CESA8*; Taylor et al., 2000; Turner and Somerville, 1997). TE specificity of the chosen *IRX1* promoter fragment (pro*IRX1*) was confirmed by detecting the pro*IRX1*-driven expression of a protein fusion between GFP and β-glucuronidase (GUS) in *Arabidopsis thaliana* seedlings (Fig. S3A-C), where positional information enables unambiguous identification of the early differentiating TEs. We created three double transgenic *MC9*-RNAi pro*IRX1*::*ATG2*-RNAi cell suspension lines (Fig. S1A,B). Because living TEs represent less than 10% of the differentiating cell cultures (e.g. Fig. 1B; data not shown), TE-specific downregulation of *ATG2*, which is expressed in both TEs and non-TEs, could not be reliably measured at the transcript level (Fig. S1A,C). However, DIC microscopy after Concanamycin A treatment revealed decreased numbers of autophagic bodies specifically in the TEs of the double RNAi lines compared to the *MC9*-RNAi alone (Fig. 5A). Most importantly, the *MC9*-RNAi ectopic cell death was fully suppressed and TE autolysis was partially restored in the *MC9*-RNAi pro*IRX1*::*ATG2*-RNAi lines (Fig. 5B), supporting that MC9-dependent regulation of autophagy fosters TE autolysis and confines cell death to the correct cells in the vascular xylem of Arabidopsis.

## DISCUSSION

Our results indicate a functional link between MC9 and autophagy during TE differentiation. The use of the TE cell culture system in which specific observation of vascular xylem cells is facilitated revealed that TEs with reduced *MC9* expression and consequently increased autophagy could be detrimental to the surrounding cells. This ectopic cell death, together with the absence of noticeable effect on the speed of TE cell death, suggest that MC9 and autophagy are not effectors of TE PCD per se. In contrast with our observations, autophagy has previously been proposed to promote xylem differentiation and to be a mechanistic component of TE PCD, based on constitutive downregulation and constitutive activation of the upstream regulator of autophagy Rab GTPase RabG3b (Kwon et al., 2010). However, it remains unclear if, where and when RabG3b is normally expressed during xylem development. Furthermore, ectopic activation or downregulation of RabG3b affects xylem differentiation rates and thus clearly affects TE differentiation at an early time point, which makes it impossible to determine the role of autophagy during later TE differentiation. We modified autophagy in a cell-type specific manner after the onset of TE differentiation, which allowed us to investigate the role of autophagy in TEs at the time of its physiologically relevant activation, and to determine the need for a tight regulation of autophagy in TEs to prevent detrimental effects on the surrounding cells.

The detrimental effect of increased autophagy and incorrect cellular autolysis of the *MC9*-RNAi TEs onto the surrounding cells suggests an incorrect release of molecules by these TEs. The TEs preparing to commit cell suicide and protoplast autolysis must build up a strong molecular arsenal that should be tightly regulated in strength, space and time to prevent harmful effects on the neighbouring cells. The importance of a strict control over the molecules released by TEs has been previously illustrated in differentiating *Zinnia elegans* cell cultures, where TEs became harmful to non-TEs when the culture medium was depleted of a protective extracellular protease inhibitor (Endo et al., 2001). However, to our knowledge, no report has since investigated the safeguards that prevent damaging of the surrounding cells by the molecular arsenal of TEs. In our TE cell suspensions, we now provide direct evidence that correct regulation of autophagy in TEs is required to foster TE autolysis and restrict cell death to the TE cell type. Therefore, autophagy seems crucial to the control of the molecules released by TEs, i.e. to intercellular signalling.

Our discovery raises the question of how an intracellular trafficking route such as autophagy can mediate intercellular communication. In mammals, a body of evidence has accumulated to imply a role for autophagy in cell-to-cell communication through its effects on some secretory pathways (for review see Bhattacharya et al., 2014). In plants, interference with intracellular trafficking routes can result in ectopic internalization of an otherwise secreted cargo (McFarlane et al., 2013), or conversely trigger the secretion of a vacuole-targeted cargo (Sanmartín et al., 2007). In particular, Kulich et al. (2013) proposed that overexpression of the Arabidopsis exocyst subunit EXO70E2 or knock-out of EXO70B1 could result in higher numbers of autophagosomes than can be targeted to the vacuole, triggering an ‘autophagic traffic jam’ and subsequent secretion of vacuole-targeted cargos. A similar situation is possible in the *MC9*-RNAi TEs where increased autophagy could lead to mistargeting of the autophagosomal cargos and altered composition of the molecules released by TEs. Alternatively, or in addition, different levels of autophagy could change the content of TEs via altered metabolism and/or vacuolar loading, and therefore affect the composition of the molecules released by dead TEs after loss of plasma membrane integrity.

A novel type of interaction was observed between a metacaspase function and autophagy during TE differentiation. Reduction in the level of *MC9* expression resulted in high levels of autophagy, meaning that full activity of MC9 is required to tune down the level of autophagy in TEs. This is different from the results obtained in spruce embryo suspensor cells where a type II metacaspase was shown to promote autophagy (Minina et al., 2013). Differing functions for the two metacaspases in the regulation of autophagy is not necessarily surprising considering that autophagy participates in several different processes during the life time of the cell. Moreover, the two metacaspases have different enzymatic properties, the spruce MC having a neutral pH optimum and MC9 having an acidic pH optimum, and therefore most probably different modes of action.

In an earlier study, METACASPASE9 was implicated in intercellular signalling that controls the spreading of cell death induced by reactive oxygen species in the leaves of *Arabidopsis thaliana* (Wrzaczek et al., 2015). In this case, MC9 acts by processing the secreted GRIM REAPER (GRI) peptide which in turn induces cell death (Wrzaczek et al., 2015). However, the aforementioned peptide was not identified within the MC9 degradome in Arabidopsis seedlings (Tsiatsiani et al., 2013). The absence of GRI in the MC9 seedling degradome suggests that GRI does not mediate the MC9-dependent intercellular communication between xylem cells. The TE cell culture system which is devoid of interference from the non-vascular cell types should be optimal to find the targets of MC9 in control of autophagy and hence intercellular signalling between the different cell types of the xylem.

In conclusion, our approach combining a simplified system with two cell types demonstrated a need for a tight control of autophagy in differentiating TEs undergoing PCD. Considering that autophagy was detected in numerous other types of PCD (see for review Galluzzi et al., 2012; Liu and Levine, 2014; Minina et al., 2014), our discoveries raise the question of whether the tight control of autophagy is a common mechanism for eukaryotic cells undergoing PCD to implement intercellular signalling for protection of the surrounding cells.

## MATERIALS AND METHODS

### Cloning

All gateway recombinations described below were performed using BP or LR clonase (11789021 and 11791100, respectively; Thermo Fisher Scientific, MD, USA).

The pro*IRX1*::*ATG2*-RNAi construct consisted of a synthesized inverted repeat of an *ATG2* (AT3G19190) coding sequence fragment (700 bp) around a 644 bp intron sub-cloned into pDONR/Zeo and further recombined into the pK2GW7 vector (Karimi et al., 2002) in which the 35S promoter was replaced by a 1586 bp fragment of *IRX1* (AT4G18780) promoter (pro*IRX1*, including 5′UTR).

The pro*IRX1*::*GFP:GUS* and *MC9*-RNAi constructs were generated by PCR amplification of the aforementioned pro*IRX1* from *Arabidopsis thaliana* gDNA and of an 85 bp *MC9* (AT5G04200) fragment from *Arabidopsis thaliana* cDNA. Next, gateway recombinations were performed into pDONR207 and pBGWFS7 (for pro*IRX1*) or pH7GWiWG2-II (for *MC9*-RNAi) vectors (Karimi et al., 2002).

### Plant material, growth conditions and induction of *in vitro* TE differentiation

Subculturing of the cell cultures and establishment of stable transgenic cell lines were performed according to Pesquet et al. (2010) except for that ten-days-old cell cultures were used for transformation and that the concentration of hygromycin (H3274; Sigma-Aldrich, MO, USA) was 12.5 µg/ml for selection of transformants.

For induction of TE differentiation, nine- to ten-days-old cell suspensions were adjusted to a density of 30 mg of cells per ml in liquid Murashige and Skoog medium (MS medium, M0222; Duchefa, Haarlem, The Netherlands,), pH 6, containing 1 mM MES (M2933; Sigma), 6 mg/l α-naphthalene acetic acid (N0640; Sigma-Aldrich), 1 mg/l 6-benzylaminopurine (B3408; Sigma-Aldrich) and 4 μM epibrassinolide (E1641; Sigma-Aldrich).

The pro*MC9*::*MC9*:GFP plants were previously described in Bollhöner et al. (2013). The pro*IRX1*::*GFP*:*GUS* plants were established using the floral dip transformation method (Clough and Bent, 1998). All plant seedlings were grown *in vitro* for four days in 16-h-daylength on solid MS medium (0.8% agar) pH 5.8 containing 1% (w/v) sucrose.

### Microscopy analyses

The stains used in all analyses were 12 nM fluorescein dictate (FDA, viability stain, F7378; Sigma-Aldrich), 15 µM presidium iodide (PI, cell wall counterstain, P4170; Sigma-Aldrich) or 4 µM Lysotracker Red DN99 (LTR, acidotropic dye, L-7528; Thermo Fisher Scientific).

To measure the proportions of TEs, TEs with remnants, living TEs and living non-TEs, cell cultures were stained with FDA and PI, and imaged with an Axioplan 2 microscope and an Axiocam HRc camera (Zeiss, Oberkochen, Germany).

For observation of vacuolar autophagic bodies: cells were either imaged as above after three hours with 10 µM Concanamycin A and with or without 1 µM wortmannin, or they were stained for two hours with LTR, then stained with FDA and imaged using Leica TCS SP2 (Leica Microsystems, Wetzlar, Germany) and Zeiss LSM780 inverted cLSM (Zeiss).

TE 3D projections were constructed by maximum intensity projection of PI-stained differentiating cell cultures imaged with a LSM780 cLSM.

pro*MC9::MC9:GFP* cell lines and pro*IRX1*::*GFP*:*GUS* seedlings were imaged with a LSM780 cLSM after PI staining. For MC9:GFP vacuolar localization, pro*MC9*::*MC9*:*GFP* seedlings were treated three hours with or without 10 µM Concanamycin A and with or without 1 µM wortmannin. Punctate quantification was performed on micrographs of single focal planes where the highest GFP fluorescence intensity was set as 100% and a 20% threshold was applied to reduce background signal.

Histochemical GUS staining of pro*IRX1*::*GFP*:*GUS* seedlings was performed according to Bollhöner et al. (2013).

### Semi-quantitative and quantitative PCR analyses

Total RNA was isolated from frozen cell homogenates using Qiagen RNeasy Plant MiniKit (74904; Qiagen, Hilden, Germany). 0.5 µg total RNA were used for cDNA synthesis using QuantiTect Reverse Transcription Kit (205313; Qiagen). qPCR reactions were run in a Roche LightCycler 480 (Roche, Basel, Switzerland) using 20 µl reactions containing 5 µl of 25 times diluted cDNA, 200 nM primers and iQ SybrGreen Supermix (1708880; Bio-Rad, CA, USA). Forty cycles (95°C, 10 s; 55°C, 20 s; 72°C, 30 s) were applied to all genes, and *MC9* (forward primer: 5′-CGACATCGGCACACATCTAC-3′; reverse primer: 5′-TGGCACCTTCATTTTCGTTC-3′) expression was normalized to two reference genes (combined by geometric mean): *UBQ10* (AT4G05320; forward primer: 5′-GGCCTTGTATAATCCCTGAT-3′; reverse primer: 5′-AAAGAGATAACAGGAACGGA-3′) and *SAND* (AT2G28390; forward primer: 5′-AACTCTATGCAGCATTTGATCCACT-3′; reverse primer: 5′-TGATTGCATATCTTTATCGCCATC-3′).

Semi-quantitative PCR reactions were also run in 20 µl containing 5 µl of 25 times diluted cDNA, 200 nM primers and Go Taq Green Master Mix (M7123; Promega Corporation, WI, USA). Using a T100 Thermal Cycler (Bio-Rad), 30 cycles (95°C, 15 s; 55°C, 15 s; 72°C, 30 s) were applied for *UBQ10* reference gene (same primers as above) while 35 cycles were applied for *MC9* (same primers as above) and for *ATG2* (forward primer: 5′-GTGCATGCTGCTGGAATCTA-3′; reverse primer: 5′-GTGTGCGGACTAATGCAGAA-3′). The corresponding products were loaded in 3% agarose gel containing 0.05% (v/v) Midori Green staining (MG-04; Nippon Genetics Europe GmbH, Germany) for electrophoresis (30 min at 100 V). Gel imaging was performed using Techtum Gel Photo System (Techtum, Sweden) and Gel-Pro Analyzer (Media Cybernetics Inc, MD, USA).

### Protein biochemistry

All analyses were performed from pools of three biological replicates.

To measure abundance of NBR1 (Svenning et al., 2011), SDS-PAGE was run using 30 µg of total proteins. The proteins were stained in gel using Coomassie Brilliant Blue R-250 (161-0435; Bio-Rad), or transferred onto a nitrocellulose membrane (Bio-Rad) for immunoblotting. The membrane was probed with 1:1000 diluted anti-NBR1 antibodies, followed by probing with 1:5000 diluted anti-rabbit horseradish peroxidase (HRP)-conjugated antibodies (sc-2301; Santa Cruz Biotechnology, Inc., TX, USA). The immunoblot was revealed using SuperSignal West Pico Chemiluminescent Substrate (Thermo Fisher Scientific) and imaged with a CCD camera (LAS-3000; FujiFilm, Japan).

### Statistical analyses

All charts displaying error bars correspond to mean values and standard deviations (s.d.) for at least three biological replicates. Data in Fig. 2D,E and Fig. 5A,B were analysed by one-way ANOVA followed by Fisher's test (two-tailed) using Minitab 17 (Cleverbridge AG, Germany). Means that do not share any letter are significantly different (*P*<0.05). For all other charts displaying error bars and comparing wild-type (or control) and *MC9*-RNAi lines (or treatments), Welch-corrected *t*-tests (two-tailed) were performed and an asterisk indicates significant difference compared to wild-type (*P*<0.05).

## References

[BIO015529C1] Alvarez V. E., Kosec G., Sant'Anna C., Turk V., Cazzulo J. J. and Turk B. (2008). Autophagy is involved in nutritional stress response and differentiation in Trypanosoma cruzi. *J. Biol. Chem.* 283, 3454-3464. 10.1074/jbc.M708474200 18039653

[BIO015529C2] Avci U., Petzold H. E., Ismail I. O., Beers E. P. and Haigler C. H. (2008). Cysteine proteases XCP1 and XCP2 aid micro-autolysis within the intact central vacuole during xylogenesis in Arabidopsis roots. *Plant J.* 56, 303-315. 10.1111/j.1365-313X.2008.03592.x 18573193

[BIO015529C3] Bhattacharya A., Prakash Y. S. and Eissa N. T. (2014). Secretory function of autophagy in innate immune cells. *Cell. Microbiol.* 16, 1637-1645. 10.1111/cmi.12365 25237740

[BIO015529C4] Blommaart E. F. C., Krause U., Schellens J. P. M., Vreeling-Sindelarova H. and Meijer A. J. (1997). The phosphatidylinositol 3-kinase inhibitors wortmannin and LY294002 inhibit autophagy in isolated rat hepatocytes. *Eur. J. Biochem.* 243, 240-246. 10.1111/j.1432-1033.1997.0240a.x 9030745

[BIO015529C5] Bollhöner B., Zhang B., Stael S., Denancé N., Overmyer K., Goffner D., Van Breusegem F. and Tuominen H. (2013). Post mortem function of AtMC9 in xylem vessel elements. *New Phytol.* 200, 498-510. 10.1111/nph.12387 23834670

[BIO015529C6] Bozhkov P. and Jansson C. (2007). Autophagy and cell-death proteases in plants: two wheels of a funeral cart. *Autophagy* 3, 136-138. 10.4161/auto.3600 17204839

[BIO015529C7] Bozhkov P. V., Suarez M. F., Filonova L. H., Daniel G., Zamyatnin A. A., Rodriguez-Nieto S., Zhivotovsky B. and Smertenko A. (2005). Cysteine protease mcII-Pa executes programmed cell death during plant embryogenesis. *Proc. Natl. Acad. Sci. USA* 102, 14463-14468. 10.1073/pnas.0506948102 16183741 PMC1242326

[BIO015529C8] Brodribb T. J. (2009). Xylem hydraulic physiology: the functional backbone of terrestrial plant productivity. *Plant Sci.* 177, 245-251. 10.1016/j.plantsci.2009.06.001

[BIO015529C9] Burgess J. and Linstead P. (1984). *In-vitro* tracheary element formation: structural studies and the effect of tri-iodobenzoic acid. *Planta* 160, 481-489. 10.1007/BF00411135 24258774

[BIO015529C10] Clough S. J. and Bent A. F. (1998). Floral dip: a simplified method for Agrobacterium-mediated transformation of Arabidopsis thaliana. *Plant J.* 16, 735-743. 10.1046/j.1365-313x.1998.00343.x 10069079

[BIO015529C11] Coll N. S., Vercammen D., Smidler A., Clover C., Van Breusegem F., Dangl J. L. and Epple P. (2010). Arabidopsis type I metacaspases control cell death. *Science* 330, 1393-1397. 10.1126/science.1194980 21097903

[BIO015529C12] Endo S., Demura T. and Fukuda H. (2001). Inhibition of proteasome activity by the TED4 protein in extracellular space: a novel mechanism for protection of living cells from injury caused by dying cells. *Plant Cell Physiol.* 42, 9-19. 10.1093/pcp/pce002 11158439

[BIO015529C13] Escamez S. and Tuominen H. (2014). Programmes of cell death and autolysis in tracheary elements: when a suicidal cell arranges its own corpse removal. *J. Exp. Bot.* 65, 1313-1321. 10.1093/jxb/eru057 24554761

[BIO015529C14] Fendrych M., Van Hautegem T., Van Durme M., Olvera-Carrillo Y., Huysmans M., Karimi M., Lippens S., Guérin C. J., Krebs M., Schumacher K. et al. (2014). Programmed cell death controlled by ANAC033/SOMBRERO determines root cap organ size in Arabidopsis. *Curr. Biol.* 24, 931-940. 10.1016/j.cub.2014.03.025 24726156

[BIO015529C15] Friedman W. E. and Cook M. E. (2000). The origin and early evolution of tracheids in vascular plants: integration of palaeobotanical and neobotanical data. *Philos. Trans. R. Soc. Lond. B Biol. Sci.* 355, 857-868. 10.1098/rstb.2000.0620 10905614 PMC1692781

[BIO015529C16] Galluzzi L., Vitale I., Abrams J. M., Alnemri E. S., Baehrecke E. H., Blagosklonny M. V., Dawson T. M., Dawson V. L., El-Deiry W. S., Fulda S. et al. (2012). Molecular definitions of cell death subroutines: recommendations of the Nomenclature Committee on Cell Death 2012. *Cell Death Differ.* 19, 107-120. 10.1038/cdd.2011.96 21760595 PMC3252826

[BIO015529C17] Groover A. and Jones A. M. (1999). Tracheary element differentiation uses a novel mechanism coordinating programmed cell death and secondary cell wall synthesis. *Plant Physiol.* 119, 375-384. 10.1104/pp.119.2.375 9952432 PMC32113

[BIO015529C18] Hackenberg T., Juul T., Auzina A., Gwiz˙dz˙ S., Małolepszy A., Van Der Kelen K., Dam S., Bressendorff S., Lorentzen A., Roepstorff P. et al. (2013). Catalase and NO CATALASE ACTIVITY1 promote autophagy-dependent cell death in Arabidopsis. *Plant Cell* 25, 4616-4626. 10.1105/tpc.113.117192 24285797 PMC3875739

[BIO015529C19] He R., Drury G. E., Rotari V. I., Gordon A., Willer M., Farzaneh T., Woltering E. J. and Gallois P. (2008). Metacaspase-8 modulates programmed cell death induced by ultraviolet light and H2O2 in Arabidopsis. *J. Biol. Chem.* 283, 774-783. 10.1074/jbc.M704185200 17998208

[BIO015529C20] Inoue Y., Suzuki T., Hattori M., Yoshimoto K., Ohsumi Y. and Moriyasu Y. (2006). AtATG genes, homologs of yeast autophagy genes, are involved in constitutive autophagy in Arabidopsis root tip cells. *Plant Cell Physiol.* 47, 1641-1652. 10.1093/pcp/pcl031 17085765

[BIO015529C21] Ito J. and Fukuda H. (2002). ZEN1 is a key enzyme in the degradation of nuclear DNA during programmed cell death of tracheary elements. *Plant Cell* 14, 3201-3211. 10.1105/tpc.006411 12468737 PMC151212

[BIO015529C22] Karimi M., Inzé D. and Depicker A. (2002). GATEWAY™ vectors for Agrobacterium-mediated plant transformation. *Trends Plant Sci.* 7, 193-195. 10.1016/S1360-1385(02)02251-3 11992820

[BIO015529C23] Kenrick P. and Crane P. R. (1997). The origin and early evolution of plants on land. *Nature* 389, 33-39. 10.1038/37918

[BIO015529C24] Klionsky D. J., Abdalla F. C., Abeliovich H., Abraham R. T., Acevedo-Arozena A., Adeli K., Agholme L., Agnello M., Agostinis P., Aguirre-Ghiso J. A. et al. (2012). Guidelines for the use and interpretation of assays for monitoring autophagy. *Autophagy* 8, 445-544. 10.4161/auto.19496 22966490 PMC3404883

[BIO015529C25] Kulich I., Pečenková T., Sekereš J., Smetana O., Fendrych M., Foissner I., Höftberger M. and Žárský V. (2013). Arabidopsis exocyst subcomplex containing subunit EXO70B1 is involved in autophagy-related transport to the vacuole. *Traffic* 14, 1155-1165. 10.1111/tra.12101 23944713

[BIO015529C26] Kuriyama H. (1999). Loss of tonoplast integrity programmed in tracheary element differentiation. *Plant Physiol.* 121, 763-774. 10.1104/pp.121.3.763 10557224 PMC59438

[BIO015529C27] Kwon S. I., Cho H. J., Jung J. H., Yoshimoto K., Shirasu K. and Park O. K. (2010). The Rab GTPase RabG3b functions in autophagy and contributes to tracheary element differentiation in Arabidopsis. *Plant J.* 64, 151-164. 10.1111/j.1365-313X.2010.04315.x 20659276

[BIO015529C28] Liu Y. and Levine B. (2014). Autosis and autophagic cell death: the dark side of autophagy. *Cell Death Differ.* 157, 65-75. 10.1038/cdd.2014.143 PMC4326571 25257169

[BIO015529C29] Lv X., Pu X., Qin G., Zhu T. and Lin H. (2014). The roles of autophagy in development and stress responses in Arabidopsis thaliana. *Apoptosis* 19, 905-921. 10.1007/s10495-014-0981-4 24682700

[BIO015529C30] McFarlane H. E., Watanabe Y., Gendre D., Carruthers K., Levesque-Tremblay G., Haughn G. W., Bhalerao R. P. and Samuels L. (2013). Cell wall polysaccharides are mislocalized to the vacuole in echidna mutants. *Plant Cell Physiol.* 54, 1867-1880. 10.1093/pcp/pct129 24058145

[BIO015529C31] Merkulova E. A., Guiboileau A., Naya L., Masclaux-Daubresse C. and Yoshimoto K. (2014). Assessment and optimization of autophagy monitoring methods in Arabidopsis roots indicate direct fusion of autophagosomes with vacuoles. *Plant Cell Physiol.* 55, 715-726. 10.1093/pcp/pcu041 24566535

[BIO015529C32] Minina E. A., Filonova L. H., Fukada K., Savenkov E. I., Gogvadze V., Clapham D., Sanchez-Vera V., Suarez M. F., Zhivotovsky B., Daniel G. et al. (2013). Autophagy and metacaspase determine the mode of cell death in plants. *J. Cell Biol.* 203, 917-927. 10.1083/jcb.201307082 24344187 PMC3871426

[BIO015529C51] Minina E. A., Bozhkov P. V. and Hofius D. (2014). Autophagy as initiator or executioner of cell death. *Trends Plant Sci.* 19, 692-697. 10.1016/j.tplants.2014.07.007 25156061

[BIO015529C33] Mizushima N. and Levine B. (2010). Autophagy in mammalian development and differentiation. *Nat. Cell Biol.* 12, 823-830. 10.1038/ncb0910-823 20811354 PMC3127249

[BIO015529C34] Mizushima N., Yamamoto A., Matsui M., Yoshimori T. and Ohsumi Y. (2004). In vivo analysis of autophagy in response to nutrient starvation using transgenic mice expressing a fluorescent autophagosome marker. *Mol. Biol. Cell* 15, 1101-1111. 10.1091/mbc.E03-09-0704 14699058 PMC363084

[BIO015529C35] Mortimore G. E. and Poso A. R. (1987). Intracellular protein catabolism and its control during nutrient deprivation and supply. *Annu. Rev. Nutr.* 7, 539-568. 10.1146/annurev.nu.07.070187.002543 3300746

[BIO015529C36] Oda Y. and Fukuda H. (2012). Initiation of cell wall pattern by a Rho- and microtubule-driven symmetry breaking. *Science* 337, 1333-1336. 10.1126/science.1222597 22984069

[BIO015529C37] Oda Y., Iida Y., Kondo Y. and Fukuda H. (2010). Wood cell-wall structure requires local 2D-microtubule disassembly by a novel plasma membrane-anchored protein. *Curr. Biol.* 20, 1197-1202. 10.1016/j.cub.2010.05.038 20619818

[BIO015529C38] Pesquet E., Korolev A. V., Calder G. and Lloyd C. W. (2010). The Microtubule-Associated Protein AtMAP70–5 regulates secondary wall patterning in Arabidopsis wood cells. *Curr. Biol.* 20, 744-749. 10.1016/j.cub.2010.02.057 20399097

[BIO015529C39] Sanmartín M., Ordóñez A., Sohn E. J., Robert S., Sánchez-Serrano J. J., Surpin M. A., Raikhel N. V. and Rojo E. (2007). Divergent functions of VTI12 and VTI11 in trafficking to storage and lytic vacuoles in Arabidopsis. *Proc. Natl. Acad. Sci. USA* 104, 3645-3650. 10.1073/pnas.0611147104 17360696 PMC1805581

[BIO015529C40] Svenning S., Lamark T., Krause K. and Johansen T. (2011). Plant NBR1 is a selective autophagy substrate and a functional hybrid of the mammalian autophagic adapters NBR1 and p62/SQSTM1. *Autophagy* 7, 993-1010. 10.4161/auto.7.9.16389 21606687 PMC3210314

[BIO015529C41] Taylor N. G., Laurie S. and Turner S. R. (2000). Multiple cellulose synthase catalytic subunits are required for cellulose synthesis in Arabidopsis. *Plant Cell* 12, 2529-2539. 10.1105/tpc.12.12.2529 11148295 PMC102235

[BIO015529C42] Thompson A. R., Doelling J. H., Suttangkakul A. and Vierstra R. D. (2005). Autophagic nutrient recycling in Arabidopsis directed by the ATG8 and ATG12 conjugation pathways. *Plant Physiol.* 138, 2097-2110. 10.1104/pp.105.060673 16040659 PMC1183398

[BIO015529C43] Torrey J. G., Fosket D. E. and Hepler P. K. (1971). Xylem formation: a paradigm of cytodifferentiation in higher plants. *Am. Sci.* 59, 338-352.

[BIO015529C44] Tsiatsiani L., Timmerman E., De Bock P.-J., Vercammen D., Stael S., Van De Cotte B., Staes A., Goethals M., Beunens T., Van Damme P. et al. (2013). The Arabidopsis metacaspase9 degradome. *Plant Cell* 25, 2831-2847. 10.1105/tpc.113.115287 23964026 PMC3784583

[BIO015529C45] Turner S. R. and Somerville C. R. (1997). Collapsed xylem phenotype of Arabidopsis identifies mutants deficient in cellulose deposition in the secondary cell wall. *Plant Cell* 9, 689-701. 10.1105/tpc.9.5.689 9165747 PMC156949

[BIO015529C46] Uren A. G., O'Rourke K., Aravind L., Pisabarro M. T., Seshagiri S., Koonin E. V. and Dixit V. M. (2000). Identification of paracaspases and metacaspases: two ancient families of caspase-like proteins, one of which plays a key role in MALT lymphoma. *Mol. Cell* 6, 961-967. 10.1016/s1097-2765(00)00094-0 11090634

[BIO015529C47] Watanabe N. and Lam E. (2011). Calcium-dependent activation and autolysis of Arabidopsis metacaspase 2d. *J. Biol. Chem.* 286, 10027-10040. 10.1074/jbc.M110.194340 21209078 PMC3060454

[BIO015529C48] Wodzicki T. J. and Brown C. L. (1973). Organization and breakdown of protoplast during maturation of pine tracheids. *Am. J. Bot.* 60, 631-640. 10.2307/2441440

[BIO015529C49] Wrzaczek M., Vainonen J. P., Stael S., Tsiatsiani L., Help-Rinta-Rahko H., Gauthier A., Kaufholdt D., Bollhöner B., Lamminmäki A., Staes A. et al. (2015). GRIM REAPER peptide binds to receptor kinase PRK5 to trigger cell death in Arabidopsis. *EMBO J.* 34, 55-66. 10.15252/embj.201488582 25398910 PMC4291480

[BIO015529C50] Yoshimoto K., Hanaoka H., Sato S., Kato T., Tabata S., Noda T. and Ohsumi Y. (2004). Processing of ATG8s, ubiquitin-like proteins, and their deconjugation by ATG4s are essential for plant autophagy. *Plant Cell* 16, 2967-2983. 10.1105/tpc.104.025395 15494556 PMC527192

